# 3-Bromo-*N*′-(2-hydr­oxy-3,5-diiodo­benzyl­idene)benzohydrazide monohydrate

**DOI:** 10.1107/S1600536809010964

**Published:** 2009-03-28

**Authors:** Jing-Heng Ning, Xiao-Wu Xu

**Affiliations:** a College of Chemical and Biological Engineering, Changsha University of Science and Technology, Changsha 410004, People’s Republic of China; b Changsha Chemical Industry Research Institute, Changsha 410007, People’s Republic of China

## Abstract

Crystals of the title compound, C_14_H_9_BrI_2_N_2_O_2_·H_2_O, were obtained from a condensation reaction of 3-bromo­benzohydrazide with 3,5-diiodo­salicylaldehyde. The Schiff base mol­ecule assumes an *E* configuration with respect to the C=N bond, and the dihedral angle between the two benzene rings is 6.9 (2)°. An intra­molecular O—H⋯N hydrogen bond is observed in the Schiff base mol­ecule and may contribute to its overall near planarity. In the crystal structure, mol­ecules are linked through inter­molecular O—H⋯O and N—H⋯O hydrogen bonds, forming layers parallel to the *bc* plane. Short inter­molecular I⋯O contacts [2.930 (5) Å] are also found, linking the mol­ecules into zigzag chains along *b*.

## Related literature

For the biological activity of Schiff bases, see: Bedia *et al.* (2006 ▶); Richardson & Bernhardt (1999 ▶); Koh *et al.* (1998 ▶); Prasad *et al.* (2007 ▶). For metal complexes of Schiff bases, see: Adams *et al.* (2000 ▶); Ainscough *et al.* (1998 ▶); Roth *et al.* (2007 ▶). For related structures, see: Fun *et al.* (2008 ▶); Butcher *et al.* (2007 ▶); Zhi & Yang (2007 ▶); Ejsmont *et al.* (2008 ▶); Yathirajan *et al.* (2007 ▶); Narayana *et al.* (2007 ▶). For bond-length data, see: Allen *et al.* (1987 ▶). For short inter­molecular I⋯O contacts, see, for example: Britton (2003 ▶).
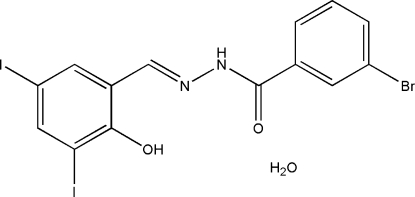


## Experimental

### 

#### Crystal data

C_14_H_9_BrI_2_N_2_O_2_·H_2_O
*M* *_r_* = 588.96
Monoclinic, 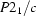

*a* = 15.181 (3) Å
*b* = 7.611 (2) Å
*c* = 15.516 (3) Å
β = 110.628 (3)°
*V* = 1677.8 (6) Å^3^
*Z* = 4
Mo *K*α radiation
μ = 6.14 mm^−1^
*T* = 298 K
0.23 × 0.20 × 0.20 mm

#### Data collection

Bruker SMART 1000 CCD area-detector diffractometer
Absorption correction: multi-scan (*SADABS*; Bruker, 2001 ▶) *T*_min_ = 0.261, *T*_max_ = 0.293
13552 measured reflections
3656 independent reflections
2651 reflections with *I* > 2σ(*I*)
*R*_int_ = 0.059

#### Refinement

*R*[*F* ^2^ > 2σ(*F* ^2^)] = 0.042
*wR*(*F* ^2^) = 0.090
*S* = 1.00
3656 reflections
209 parameters
4 restraints
H atoms treated by a mixture of independent and constrained refinement
Δρ_max_ = 0.63 e Å^−3^
Δρ_min_ = −0.69 e Å^−3^

### 

Data collection: *SMART* (Bruker, 2007 ▶); cell refinement: *SAINT* (Bruker, 2007 ▶); data reduction: *SAINT*; program(s) used to solve structure: *SHELXTL* (Sheldrick, 2008 ▶); program(s) used to refine structure: *SHELXTL*; molecular graphics: *SHELXTL*; software used to prepare material for publication: *SHELXTL*.

## Supplementary Material

Crystal structure: contains datablocks global, I. DOI: 10.1107/S1600536809010964/sj2600sup1.cif

Structure factors: contains datablocks I. DOI: 10.1107/S1600536809010964/sj2600Isup2.hkl

Additional supplementary materials:  crystallographic information; 3D view; checkCIF report

## Figures and Tables

**Table 1:** Hydrogen-bond geometry (Å, °)

*D*—H⋯*A*	*D*—H	H⋯*A*	*D*⋯*A*	*D*—H⋯*A*
O3—H3*A*⋯O1^i^	0.86 (4)	2.50 (6)	3.131 (6)	132 (6)
N2—H2⋯O3^ii^	0.89 (4)	2.08 (6)	2.934 (6)	162 (7)
O1—H1⋯N1	0.82	1.87	2.579 (6)	144

Symmetry codes: (i) 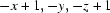; (ii) 
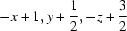
.

**Fig. 1.**
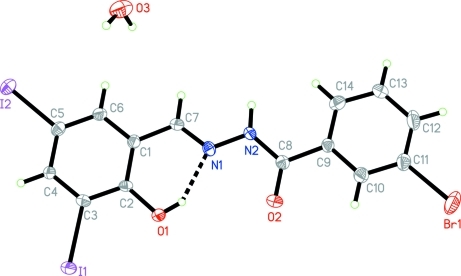
The molecular structure of (I), with 30% probability displacement ellipsoids. The intramolecular hydrogen bond is shown as a dashed line.

**Fig. 2.**
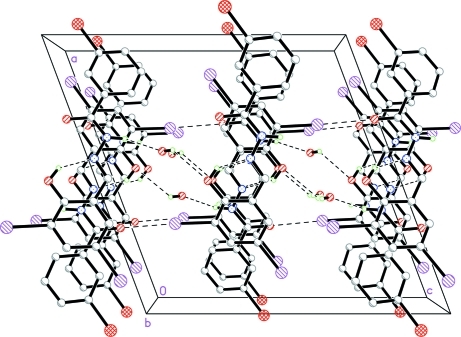
Molecular packing of (I), viewed along the b axis. H atoms not involved in the interactions have been omitted for clarity. Intermolecular hydrogen bonds and short I···O contacts are shown as dashed lines.

## supplementary crystallographic information

### Comment

Schiff bases have been demonstrated to possess interesting biological activities (Bedia *et al.*,  2006; Richardson & Bernhardt, 1999; Koh *et al.*,  1998; Prasad *et al.*, 2007). These compounds have been widely used as versatile ligands in coordination chemistry (Adams *et al.*, 2000; Ainscough *et al.*,  1998; Roth *et al.*, 2007). Recently, the crystal structures of such compounds have been extensively reported (Fun *et al.*, 2008; Butcher *et al.*,  2007; Zhi & Yang, 2007). In this paper, the new title Schiff base, (I), Fig. 1, is reported.

The asymmetric unit of (I) contains a Schiff base molecule and a water molecule of crystallization. The Schiff base molecule assumes an E configuration with respect to the C═N bond. The dihedral angle between the two benzene rings is 6.9 (2)°, indicating that the molecule is essentially planar. An intramolecular O—H···N hydrogen bond is observed in the Schiff base molecule and may contribute to its overall planarity. All bond lengths in (I) are within normal ranges (Allen *et al.*, 1987) and comparable to the corresponding values in other similar compounds (Ejsmont *et al.*,  2008; Yathirajan *et al.*,  2007; Narayana *et al.*,  2007).

In the crystal structure, molecules are linked through intermolecular O–H···O and N–H···O (Table 1) hydrogen bonds, forming layers parallel to the bc plane (Fig. 2).  Additional short intermolecular I1···O12^i^ contacts, 2.930 (5)Å, ^i^ = 1-x, -1/2+y, 1/2+z, are also observed linking molecules into zig-zag chains along *b*.  Similar short I···O contacts have been reported previously (Britton, 2003).

### Experimental

3-Bromobenzohydrazide (1.0 mmol, 215.2 mg) and 3,5-diiodosalicylaldehyde (1.0 mmol, 374.9 mg) were stirred at room temperature for two hours. The filtrate was kept in air for a week to obtain yellow block-shaped crystals of (I).

### Refinement

Atoms H2, H3A and H3B were located in a difference Fourier map and refined isotropically, with the N–H, O–H, and H···H distances restrained to 0.90 (1), 0.85 (1), and 1.37 (2) Å, respectively. Other H atoms were positioned geometrically and refined using a riding model with d(C–H) = 0.93 Å, d(O–H) = 0.82 Å and *U*_iso_ = 1.2*U*_eq_(C) and 1.5*U*_eq_(O).

### Crystal data

**Table:**

C_14_H_9_BrI_2_N_2_O_2_·H_2_O	*F*(000) = 1096
*M**_r_* = 588.96	*D*_x_ = 2.331 Mg m^−^^3^
Monoclinic, *P*2_1_/*c*	Mo *K*α radiation, λ = 0.71073 Å
Hall symbol: -P 2ybc	Cell parameters from 2004 reflections
*a* = 15.181 (3) Å	θ = 2.6–24.5°
*b* = 7.611 (2) Å	µ = 6.14 mm^−^^1^
*c* = 15.516 (3) Å	*T* = 298 K
β = 110.628 (3)°	Block, yellow
*V* = 1677.8 (6) Å^3^	0.23 × 0.20 × 0.20 mm
*Z* = 4	

### Data collection

**Table:**

Bruker SMART 1000 CCD area-detector diffractometer	3656 independent reflections
Radiation source: fine-focus sealed tube	2651 reflections with *I* > 2σ(*I*)
graphite	*R*_int_ = 0.059
ω scans	θ_max_ = 27.0°, θ_min_ = 1.4°
Absorption correction: multi-scan (*SADABS*; Bruker, 2001)	*h* = −19→19
*T*_min_ = 0.261, *T*_max_ = 0.293	*k* = −9→9
13552 measured reflections	*l* = −19→19

### Refinement

**Table:**

Refinement on *F*^2^	Primary atom site location: structure-invariant direct methods
Least-squares matrix: full	Secondary atom site location: difference Fourier map
*R*[*F*^2^ > 2σ(*F*^2^)] = 0.042	Hydrogen site location: inferred from neighbouring sites
*wR*(*F*^2^) = 0.090	H atoms treated by a mixture of independent and constrained refinement
*S* = 1.00	*w* = 1/[σ^2^(*F*_o_^2^) + (0.0296*P*)^2^] where *P* = (*F*_o_^2^ + 2*F*_c_^2^)/3
3656 reflections	(Δ/σ)_max_ = 0.001
209 parameters	Δρ_max_ = 0.63 e Å^−^^3^
4 restraints	Δρ_min_ = −0.69 e Å^−^^3^

### Special details

**Table:**

Geometry. All esds (except the esd in the dihedral angle between two l.s. planes) are estimated using the full covariance matrix. The cell esds are taken into account individually in the estimation of esds in distances, angles and torsion angles; correlations between esds in cell parameters are only used when they are defined by crystal symmetry. An approximate (isotropic) treatment of cell esds is used for estimating esds involving l.s. planes.
Refinement. Refinement of F^2^ against ALL reflections. The weighted R-factor wR and goodness of fit S are based on F^2^, conventional R-factors R are based on F, with F set to zero for negative F^2^. The threshold expression of F^2^ > 2sigma(F^2^) is used only for calculating R-factors(gt) etc. and is not relevant to the choice of reflections for refinement. R-factors based on F^2^ are statistically about twice as large as those based on F, and R- factors based on ALL data will be even larger.

### Fractional atomic coordinates and isotropic or equivalent isotropic displacement parameters (Å^2^)

**Table:**

	*x*	*y*	*z*	*U*_iso_*/*U*_eq_	
I1	0.32291 (3)	−0.01495 (5)	0.20582 (3)	0.04369 (14)	
I2	0.14532 (3)	−0.04420 (6)	0.49489 (3)	0.04849 (15)	
Br1	1.04870 (5)	0.64844 (11)	0.62538 (6)	0.0699 (3)	
O1	0.4774 (3)	0.1800 (5)	0.3704 (2)	0.0362 (9)	
H1	0.5212	0.2190	0.4140	0.054*	
O2	0.6948 (3)	0.4091 (6)	0.4847 (3)	0.0465 (11)	
O3	0.4178 (4)	0.0028 (7)	0.7467 (3)	0.0636 (14)	
N1	0.5549 (3)	0.3106 (5)	0.5331 (3)	0.0303 (10)	
N2	0.6303 (3)	0.3938 (6)	0.5939 (3)	0.0318 (10)	
C1	0.4070 (4)	0.1717 (7)	0.4882 (4)	0.0301 (12)	
C2	0.4058 (4)	0.1324 (6)	0.3995 (3)	0.0260 (11)	
C3	0.3290 (4)	0.0455 (7)	0.3393 (4)	0.0314 (12)	
C4	0.2549 (4)	−0.0061 (7)	0.3655 (4)	0.0312 (13)	
H4	0.2040	−0.0660	0.3244	0.037*	
C5	0.2572 (4)	0.0324 (7)	0.4540 (4)	0.0333 (13)	
C6	0.3317 (4)	0.1209 (7)	0.5138 (4)	0.0325 (13)	
H6	0.3323	0.1476	0.5724	0.039*	
C7	0.4852 (4)	0.2632 (7)	0.5544 (4)	0.0323 (13)	
H7	0.4844	0.2872	0.6129	0.039*	
C8	0.7013 (4)	0.4386 (7)	0.5641 (4)	0.0304 (12)	
C9	0.7872 (4)	0.5200 (6)	0.6326 (4)	0.0301 (12)	
C10	0.8620 (4)	0.5467 (7)	0.6036 (4)	0.0389 (14)	
H10	0.8576	0.5140	0.5444	0.047*	
C11	0.9424 (4)	0.6211 (8)	0.6616 (4)	0.0429 (15)	
C12	0.9500 (4)	0.6747 (9)	0.7478 (5)	0.0530 (17)	
H12	1.0052	0.7269	0.7863	0.064*	
C13	0.8755 (4)	0.6511 (8)	0.7772 (4)	0.0489 (16)	
H13	0.8802	0.6884	0.8357	0.059*	
C14	0.7934 (5)	0.5721 (7)	0.7204 (4)	0.0422 (15)	
H14	0.7433	0.5543	0.7407	0.051*	
H2	0.626 (5)	0.411 (9)	0.649 (2)	0.080*	
H3A	0.414 (6)	−0.082 (4)	0.709 (3)	0.080*	
H3B	0.414 (5)	0.097 (3)	0.717 (3)	0.080*	

### Atomic displacement parameters (Å^2^)

**Table:**

	*U*^11^	*U*^22^	*U*^33^	*U*^12^	*U*^13^	*U*^23^
I1	0.0401 (3)	0.0640 (3)	0.0293 (2)	−0.00844 (19)	0.01519 (18)	−0.01025 (18)
I2	0.0423 (3)	0.0602 (3)	0.0522 (3)	−0.0090 (2)	0.0281 (2)	−0.0014 (2)
Br1	0.0381 (4)	0.0888 (6)	0.0902 (6)	−0.0056 (4)	0.0317 (4)	0.0133 (5)
O1	0.029 (2)	0.054 (3)	0.027 (2)	−0.0065 (19)	0.0114 (18)	−0.0023 (18)
O2	0.047 (3)	0.067 (3)	0.025 (2)	−0.009 (2)	0.013 (2)	−0.004 (2)
O3	0.062 (3)	0.079 (3)	0.058 (3)	0.014 (3)	0.031 (3)	0.017 (3)
N1	0.024 (2)	0.032 (2)	0.030 (3)	0.0035 (19)	0.003 (2)	0.000 (2)
N2	0.026 (3)	0.042 (3)	0.026 (3)	−0.005 (2)	0.007 (2)	−0.005 (2)
C1	0.027 (3)	0.032 (3)	0.029 (3)	−0.001 (2)	0.008 (2)	0.004 (2)
C2	0.028 (3)	0.030 (3)	0.021 (3)	0.004 (2)	0.009 (2)	0.003 (2)
C3	0.037 (3)	0.033 (3)	0.026 (3)	0.001 (3)	0.012 (3)	−0.003 (2)
C4	0.025 (3)	0.041 (3)	0.027 (3)	−0.006 (2)	0.009 (2)	−0.002 (2)
C5	0.030 (3)	0.035 (3)	0.037 (3)	0.003 (2)	0.014 (3)	0.006 (2)
C6	0.035 (3)	0.035 (3)	0.031 (3)	0.000 (2)	0.015 (3)	−0.001 (2)
C7	0.033 (3)	0.039 (3)	0.024 (3)	−0.001 (3)	0.010 (3)	0.000 (2)
C8	0.030 (3)	0.029 (3)	0.029 (3)	0.002 (2)	0.006 (3)	0.004 (2)
C9	0.028 (3)	0.029 (3)	0.031 (3)	−0.003 (2)	0.008 (3)	0.004 (2)
C10	0.039 (4)	0.038 (3)	0.039 (3)	−0.005 (3)	0.013 (3)	0.001 (3)
C11	0.032 (3)	0.048 (4)	0.045 (4)	−0.001 (3)	0.009 (3)	0.009 (3)
C12	0.031 (4)	0.062 (4)	0.053 (4)	−0.009 (3)	0.000 (3)	0.011 (3)
C13	0.048 (4)	0.065 (4)	0.031 (3)	−0.010 (3)	0.011 (3)	−0.002 (3)
C14	0.046 (4)	0.044 (4)	0.042 (4)	−0.002 (3)	0.022 (3)	0.003 (3)

### Geometric parameters (Å, °)

**Table:**

I1—C3	2.092 (5)	C4—C5	1.394 (7)
I2—C5	2.094 (6)	C4—H4	0.9300
Br1—C11	1.898 (6)	C5—C6	1.362 (7)
O1—C2	1.364 (6)	C6—H6	0.9300
O1—H1	0.8200	C7—H7	0.9300
O2—C8	1.222 (6)	C8—C9	1.496 (7)
O3—H3A	0.86 (4)	C9—C10	1.377 (8)
O3—H3B	0.84 (3)	C9—C14	1.390 (8)
N1—C7	1.267 (6)	C10—C11	1.359 (8)
N1—N2	1.356 (6)	C10—H10	0.9300
N2—C8	1.357 (7)	C11—C12	1.363 (9)
N2—H2	0.89 (4)	C12—C13	1.373 (8)
C1—C6	1.390 (7)	C12—H12	0.9300
C1—C2	1.403 (7)	C13—C14	1.384 (8)
C1—C7	1.445 (7)	C13—H13	0.9300
C2—C3	1.380 (7)	C14—H14	0.9300
C3—C4	1.379 (7)		
			
C2—O1—H1	109.5	N1—C7—C1	120.3 (5)
H3A—O3—H3B	107 (3)	N1—C7—H7	119.9
C7—N1—N2	121.9 (5)	C1—C7—H7	119.9
N1—N2—C8	117.2 (4)	O2—C8—N2	120.4 (5)
N1—N2—H2	114 (5)	O2—C8—C9	122.2 (5)
C8—N2—H2	129 (5)	N2—C8—C9	117.4 (5)
C6—C1—C2	119.5 (5)	C10—C9—C14	120.0 (5)
C6—C1—C7	118.9 (5)	C10—C9—C8	116.2 (5)
C2—C1—C7	121.5 (5)	C14—C9—C8	123.7 (5)
O1—C2—C3	119.0 (4)	C11—C10—C9	119.8 (6)
O1—C2—C1	122.1 (5)	C11—C10—H10	120.1
C3—C2—C1	118.8 (5)	C9—C10—H10	120.1
C4—C3—C2	121.3 (5)	C10—C11—C12	121.4 (6)
C4—C3—I1	118.2 (4)	C10—C11—Br1	120.5 (5)
C2—C3—I1	120.5 (4)	C12—C11—Br1	118.1 (5)
C3—C4—C5	119.4 (5)	C11—C12—C13	119.4 (6)
C3—C4—H4	120.3	C11—C12—H12	120.3
C5—C4—H4	120.3	C13—C12—H12	120.3
C6—C5—C4	120.0 (5)	C12—C13—C14	120.5 (6)
C6—C5—I2	120.0 (4)	C12—C13—H13	119.7
C4—C5—I2	120.0 (4)	C14—C13—H13	119.7
C5—C6—C1	120.9 (5)	C13—C14—C9	118.8 (6)
C5—C6—H6	119.5	C13—C14—H14	120.6
C1—C6—H6	119.5	C9—C14—H14	120.6

### Hydrogen-bond geometry (Å, °)

**Table:**

*D*—H···*A*	*D*—H	H···*A*	*D*···*A*	*D*—H···*A*
O3—H3A···O1^i^	0.86 (4)	2.50 (6)	3.131 (6)	132 (6)
N2—H2···O3^ii^	0.89 (4)	2.08 (6)	2.934 (6)	162 (7)
O1—H1···N1	0.82	1.87	2.579 (6)	144

Symmetry codes: (i) −*x*+1, −*y*, −*z*+1; (ii) −*x*+1, *y*+1/2, −*z*+3/2.
